# The psychological results of 438 patients with persisting GERD symptoms by Symptom Checklist 90-Revised (SCL-90-R) questionnaire: Retraction

**DOI:** 10.1097/MD.0000000000010768

**Published:** 2018-06-01

The article, “The psychological results of 438 patients with persisting GERD symptoms by Symptom Checklist 90-Revised (SCL-90-R) questionnaire”,^[1]^ which appeared in Volume 97, Issue 5 of *Medicin*e, is being retracted due to plagiarism. The article was submitted to multiple journals and also published as “Psychological Results of 438 Patients with persisting Gastroesophageal Reflux Disease Symptoms by Symptom Checklist 90-Revised Questionnaire”^[2]^ in Volume 7, Issue 2 of *Euroasian Journal of Hepato-Gastroenterlogy*.
